# Levels of serum thyroxine, triidothyronine and thyrotropin in patients with acute myocardial infarction

**DOI:** 10.12669/pjms.344.14705

**Published:** 2018

**Authors:** Rukhsana Gulzar, Mulazim Hussain Bukhari, Rehma Dar, Hira Sajjad

**Affiliations:** 1 Dr. Rukhsana Gulzar, MBBS, M.Phil. Red Crescent Medical College, Lahore, Pakistan; 2 Prof. Mulazim Hussain Bukhari, MBBS, CHPE, DCP, M.Phil, FCPS, PhD, University College of Medicine, University of Lahore, Lahore, Pakistan; 3 Dr. Rehma Dar, MBBS, M.Phil. Assistant Prof. of Pathology, King Edward Medical University, Lahore, Pakistan; 4 Dr. Hira Sajjad, Third year MBBS. Rahbar Medical and Dental College, Lahore, Pakistan

**Keywords:** Acute myocardial infarction, Thyroid stimulating Hormone, Thyroxine, Triiodothyronine, Thyrotropin

## Abstract

**Objective:**

To determine the levels of serum thyroxine (T3, T4), triiodothyronine and thyrotropin in patients with acute myocardial infarction (AMI).

**Methods:**

It was an observational prospective study and 60 patients (both males and females) of AMI, (diagnosed by ECG & cardiac enzymes.) were included. Serum thyroid stimulating hormone (TSH), Free T3 and Free T4 were measured and relevant data was entered into a predesigned proforma.

**Results:**

FT3 levels were below the reference range in 56.7% cases while both the FT4 and TSH values were found to be the lower limit of normal range. When the decrease in FT3 was correlated with the duration of illness, it was found that significant inverse correlation existed between FT3 value and the duration of illness and linear regression line was obtained. No such correlation existed between FT4 and TSH values.

**Conclusion:**

Thyroid hormone levels (FT3) decreases in AMI and this change is associated with the duration of illness.

## INTRODUCTION

The thyroid gland is responsible for modulating several bodily functions. By manufacturing the correct quantity of thyroid hormones, it helps the body’s metabolism, the musculoskeletal performance and also the traditional integrity of the skin.

It has some effect on almost every organ of the body including the heart and many other systems. Thyroid hormones increase heat production, increase gas consumption and increase the quantity of β adrenergic receptors. Clinically, patients with enlarged levels of thyroid hormones have symptoms of enlarged metabolism whereas those with low levels of thyroid hormones exhibit symptoms of low metabolism.^1^

In a variety of non-thyroidal illnesses (NTIs) and in those undergoing surgery or fasting, thyroid hormone levels become abnormal in the absence of pituitary or thyroidal dysfunction. Euthyroid Sick Syndrome (ESS) is the term used to identify these abnormalities in thyroid function tests.^2^ Patients with NTIs are clinically euthyroid but have low circulatory concentration of total and absolute free T3, low or normal total T4, elevated concentration of absolute FT4 and normal or subnormal levels of TSH.

Chopra et al. have classified the patterns of abnormalities of thyroid hormone levels in ESS into four major types namely low T3 syndrome, low T3 and low T4 syndrome, high T4 syndrome, other variants.^3^

Low T3 Syndrome is defined as a condition in which T3 is decreased but T4 and TSH levels remain normal due to the impaired conversion of the inactive pro-hormone T4 to the biologically active hormone T3 by 5 monodeiodinase in the liver.^4^ It is the most common abnormality amongst NTIs observed. Low T3 Syndrome is associated with the inhibition of 5 monodeiodinase, an enzyme which converts T4 to the active metabolite T3 in the peripheral tissues^5^

In ESS, alterations in thyroid function occur due to complex mechanisms. Changes may occur at all levels of hypothalamic pituitary thyroid axis.^5^ Multiple factors are responsible for these changes including alteration in Type 1 and 3 deiodinase activity, thyrotropin releasing hormone and thyroid stimulating hormone secretion, hormone binding to plasma protein, thyroid hormone transporter expression and activity and thyroid hormone nuclear receptor complex. The major cause of these hormonal changes is the release of cytokines. As many as 3% of hospitalized patients have subnormal TSH values on admission which is often associated with acute phase of illness, or with glucocorticoids, or dopamine therapy.^6^,^7^

The exact cause of these changes remains controversial and undermined. They are associated with the severity of the underlying illness and disappear with recovery from the illness. Low levels of thyroid hormones predict a poor prognosis.^2^

The New York Heart Association functional classification states that the severity of heart disease is proportional to the decrease in T3 level.^8^ Results of some cross-sectional studies of patients undergoing coronary angiography suggest that free thyroxine or free triiodothyronine level was inversely and thyroid stimulating hormone concentration was positively associated with the presence of CHD or the severity of coronary atherosclerosis in euthyroid subjects.^9^-^13^ In HUNT study- a prospective population based cohort study in Norway, it was observed that low thyroid function within the clinically normal range was associated with increased mortality from CHD in women during 12-year follow-up.^1^ More studies are needed to examine the relationship between thyroid function and CHD in euthyroid individuals.^14^,^15^

The objective of this study was to measure serum thyroxine, triiodothyronin and thyrotropin levels in patients with acute myocardial infarction.

## METHODS

It was an observational prospctive study conducted at PGMI, Lahore, after approval of Institutional Ethical Review Committee and Advance studies and Research board of University of Health Sciences (UHS), Lahore. The study included patients of all ages and both genders, with the diagnosis of AMI, admitted to the CCU of Jinnah Hospital, Lahore. Diagnosis of AMI was established on the basis of ECG and cardiac enzymes.

Patients with known or suspected thyroid dysfunction, symptoms and signs of hypothyroidism and hyperthyroidism and patients using thyroid hormone or anti thyroid medication were excluded from the study.

The 60 patients were included in the study. After obtaining the informed consent of all the subjects, the personal information of the subjects was recorded on the prescribed proforma. Samples from twelve patients were drawn between 1-6 hours of onset of symptoms. Forty one samples were drawn between 7-18 hours of onset of symptoms. Samples from six were drawn after approximately 18 hours of onset of symptoms.

The 5 ml of venous blood was collected in disposable syringe. Blood was allowed to clot and centrifuged. Serum was preserved for TSH, FT3 and FT4 estimation. “Biocheck kits” were used to assay T3, T4 and TSH. The specimens were run in two batches for each assay. Each batch consisted of 30 patient samples, two controls and 6 standards.

Sample size of 60 patients was calculated by using 5% level of error with expected level of T3 1.23+0.25 ng/ml at the time of admission in AMI patients. This estimation yielded 95% level of confidence. This estimation was done by Pear T test procedure in power and precision 3.0.^16^ Software SPSS version 18.0 was used for the statistical analysis of the data gathered.

## RESULTS

Fifty-two out of sixty patients (86.67%) were male and 8 (13.33%) patients were female. Mean age for male patients was 51.7 ± 13.1 and for female patients was 58.6 ± 10.3 years. This difference in age was not found statistically significant p>0.05 (Fig.1).

**Fig.I:**
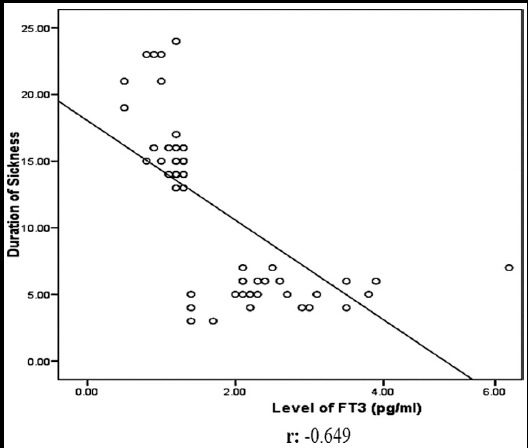
Regression line between FT3 level and the duration of illness. (Note r= -0.649).

Among male patients, mean FT3 was found to be 1.9 ± 1.22 pg/ml. This shows that a number of subjects were below the normal range of 1.4-4.2 pg/ml. Almost in every age group nearly half the subjects fell below the reference range; however, 24/60 (40%) patients were in the age group of 55-69 years, whom FT3 was found below the reference range. Similar results were obtained in female patients. Mean value of FT3 in female groups (1.7 ± 0.8 pg/ml) was not significantly different from the mean value of the male groups. However the reference range of FT4 (0.8-2.0 ng/ml) was not seemed to be disturbed in any age group both in males and females. Although all FT4 values were found to be in the reference range, these values were closer to the lower limit of reference ranges (Table I and II).

**Table-I:** Mean±SD of FT3, FT4 and TSH of Cases of AMI by Age and Gender.

Age (years)	FT3		FT4		TSH	
	
	Male	Female	Male	Female	Male	Female
25-39	2.4 ± 1.8	0	1.7 ± 0.4	0	1.4 ± 1.0	0
40-54	2.0 ± 1.0	2.0 ± 1.0	1.5 ± 0.4	1.4 ± 0.2	1.3 ± 1.3	0.6 ± 0.1
55-69	1.7 ± 0.9	1.5 ± 0.8	1.5 ± 0.3	1.4 ± 0.2	1.4 ± 1.1	1.1 ± 1.4
>70years	1.2 ± 0.2	1.8 ± 1.0	1.4 ± 0.6	1.6 ± 0.1	1.9 ± 1.3	1.4 ± 1.1
Mean ± SD	1.9 ± 1.3	1.7 ± 0.8	1.5 ± 0.4	1.4 ± 0.2	1.4 ± 1.1	1.0 ± 1.1

**Table-II:** Mean±SD of FT3, FT4 and TSH of Cases of AMI by Age and Gender.

Age (years)	FT3 (1.4-4.2pg/ml)		FT4 (0.8-2.0 ng/ml)		TSH (0.4-6.0uIU/mL)	

	Male	Female	Male	Female	Male	Female
25-39	2.4 ± 1.8	0	1.7 ± 0.4	0	1.4 ± 1.0	0
40-54	2.0 ± 1.0	2.0 ± 1.0	1.5 ± 0.4	1.4 ± 0.2	1.3 ± 1.3	0.6 ± 0.1
55-69	1.7 ± 0.9	1.5 ± 0.8	1.5 ± 0.3	1.4 ± 0.2	1.4 ± 1.1	1.1 ± 1.4
>70years	1.2 ± 0.2	1.8 ± 1.0	1.4 ± 0.6	1.6 ± 0.1	1.9 ± 1.3	1.4 ± 1.1
Mean ± SD	1.9 ± 1.3	1.7 ± 0.8	1.5 ± 0.4	1.4 ± 0.2	1.4 ± 1.1	1.0 ± 1.1

The overall status of FT3 was classified into three groups i.e. reference range (1.4-4.2 pg/ml), low (<1.4 pg/ml) and high (>4.2pg/ml). Thirty four (56.7%) patients had moderately low FT3 level.

Strong negative correlation was found between FT3 level and the duration of sickness. The coefficient of correlation (r value) was -0.65.Only three (5.0%) cases had FT4 level below the reference range. Fifty five (91.7%) out of sixty cases had serum FT4 within the reference range and only two (3.3%) were found to be above the reference range (Table-I). Like serum FT3, there was no statistically significant difference for FT4 between the two genders.

Fifty six (93.3%) cases had TSH levels within the reference range. None of the cases had higher than reference and only four (6.6%) had TSH levels below the reference range (Table-I).In the current study, considering the duration of sickness, almost half (51.7%) cases were having symptoms for 12-18 hours. Few cases (11.0%) had duration of sickness greater than 18 hours. Similarly few cases (20.0%) reported in the first six hours of the onset of symptoms (Table-III).

**Table-III:** Distribution of cases of AMI by FT3 level and gender.

Range of FT3	Male		Female		Total	Mean±SD	p-Value

	n	Mean±SD	n	Mean±SD			
Low (<1.4pg/ml)	29	1.1±0.3	5	1.0± 0.6	34 (56.7%)	1.1±0.2	<0.001
Normal (1.4-4.2pg/ml)	22	2.7±0.7	3	2.6±0.9	25 (41.7%)	2.7±0.7	
High (>4.2pg/ml)	1	6.9±0.0	0	0	1 (1.7%)	9±0.0	
Total	52	1.9±0.6	8	1.7±0.8	60	1.97±0.4	

***Note:*** There was no statistically significant difference for FT3 values between the two genders (Table-II).

In the majority of cases (60.0%), either no accompanying disease was present or no record was available. In others eleven (18.3%) patients had hypertension, only six (10.0% had diabetes mellitus and in seven (11.7%) cases both diabetes mellitus and hypertension were present.

## DISCUSSION

In the current study, significant correlation was found, between FT3 level and acute myocardial infarction, p<0.001, indicating that serum FT3 level decreases in patients with acute myocardial infarction. This study confirms other epidemiological studies which demonstrate that thyroid hormones are down regulated in any acute illness in otherwise euthyroid subjects. Although FT3 was lower in the majority of subjects this value was found to be close to the normal range and subjects having extremely low values were very few. This pattern resembles low T3 syndrome pattern of NTI, which is the most common hormonal abnormality found in almost 70% of hospitalized patients.^2^ In one study T3 levels were decreased in 85% of patients.^14^,^17^

In several cross sectional studies it was observed that low T3 syndrome was found in up to 30% of patients with congestive heart failure.^9^ Enia et al. found that low levels of serum T3 is the most common disturbance found in thyroid function. In recent years, accumulating evidence has revealed that the “low triiodothyronine” syndrome is a strong prognostic, independent predictor of death in patients affected by both acute and chronic heart disease. Approximately one fourth of patients with end stage renal disease have low FT3.^18^

In this study only FT3 was reduced, however FT4 and TSH were not grossly altered in any age group. Only three patients had decreased FT4 and only four patients had decreased TSH level. Serum TSH concentrations are usually within reference range but may be mild to moderately depressed during the acute phase of non-thyroidal illness, or slightly elevated during recovery from a severe illness.^8^ TSH might be affected because of glucocorticoid, dopamine, altered nutrition or altered biological activity of immunoreactive TSH.^6^-^8^

**Table-IV:** Distribution of cases of AMI by the duration of sickness.

Time since onset of symptoms (in hours)	No. of Cases	Percentage

	N	%
01-06 hrs	12	20
07-12 hrs	10	16.7
13-18 hrs	31	51.7
>18hrs	7	11.6
Total	60	100

Current evidence shows that T3 levels significantly decline after myocardial infarction (MI) both in animal models and in patients due to the reduced conversion of T4 into T3, accompanied by increased conversion of T4 into the inactive rT3 by the up regulation of Type-3 deiodinase.^17^ Several studies have shown that the low T3 syndrome may have an adverse prognostic impact on various acute and chronic cardiac disorders.^19^ Importantly, many of the cardiac alterations observed in subclinical hypothyroidism are reversed once thyroid function has been normalized.^20^

In this study one patient had FT3 level of 6.9pg/ml which was more than the upper limit of reference range. His FT4 was also higher than the normal and TSH was decreased, suggesting that the patient might be a case of hyperthyroidism. Sometimes chest pain and ECG changes suggestive of ischaemia can be the presenting features of hyperthyroidism. These symptoms revert with successful treatment of hyperthyroidism.^21^ In the elderly patients with underlying coronary artery disease, this is due to the increase in myocardial O_2_ demand due to the increase in cardiac contractility and work load which is associated with thyrotoxicosis. Atrial fibrillation is frequently seen in thyrotoxicosis, but sinus tachycardia is the most common rhythm disturbance recorded in almost all patients with hyperthyroidism.^22^

All the seven patients who had samples drawn after 18hours of onset of symptoms and twenty seven patients who had samples taken at 12-18 hours of onset of symptoms had low T3 levels. This confirms other studies which show that in AMI thyroid hormone level is rapidly down regulated. These studies further suggest that maximum changes occur in 24-36 hours after the onset of symptoms.^22^

The exact cause of changes in thyroid hormone levels observed in ESS remains controversial and undermined. Low T3 is considered to be the result of pathological conditions and malabsorptions that tends to decrease the survival rather than a physiological adaptation to conserve the energy stores of the body.^23^

## CONCLUSION

In this study, we found that thyroid function is depressed in AMI. Inverse correlation exists between thyroid function and the duration of illness, but no correlation exists with age or gender of the patients. T3 level is depressed but T4 and TSH levels remained within their respective reference ranges, resembling the low T3 syndrome pattern of ESS. Low levels of thyroid hormones predict poor prognosis in severe illnesses, and the use of thyroid hormone therapy is still controversial. Controlled trials with large sample size can be carried out to assess the benefits of thyroid hormone replacement therapy.

### Recommendations

Future clinical and experimental studies need to investigate the low thyroid profiles, more deeply during an AMI event to completely understand its pathophysiology and recognize whether it has a potential prognostic role for a subgroup of AMI patients or it manifests as an “epiphenomenon” due to critical illness. On the basis of our study, if physicians agree they may include these hormones in the investigations of AMI
